# Performance Evaluation of the Polyurethane-Based Composites Prepared with Recycled Polymer Concrete Aggregate

**DOI:** 10.3390/ma13030616

**Published:** 2020-01-30

**Authors:** Wenbo Ma, Zenggang Zhao, Shuaicheng Guo, Yanbing Zhao, Zhiren Wu, Caiqian Yang

**Affiliations:** 1 College of Civil Engineering and Mechanics, Xiangtan University, Xiangtan 411105, China; mawenbo@xtu.edu.cn (W.M.); 201721572083@smail.xtu.edu.cn (Z.Z.); 2 Key Laboratory of Building Safety and Energy Efficiency of the Ministry of Education, Hunan University, Changsha 410082, China; 3 College of Civil Engineering, Southeast University, Nanjing 211189, China; ybzhao@seu.edu.cn; 4 School of the Environment and Safety Engineering, Jiangsu University, Zhenjiang 212013, China; wwu5353@j-c-k.co.jp

**Keywords:** W-OH polyurethane, Pisha sandstone, ethylene-vinyl acetate (EVA), wet-dry cycles, freeze-thaw cycles, unconfined compressive strength, triaxle compressive strength

## Abstract

Currently the investigation on recycled cement concrete aggregate has been widely conducted, while the understanding of the recycled polymer concrete aggregate is still limited. This study aims to fill this knowledge gap through the experimental investigation on mechanical and durability performance. Specifically, the remolded polyurethane stabilized Pisha sandstone was collected as the recycled polymer concrete aggregate. The remolded Pisha sandstone was then applied to re-prepare the polyurethane-based composites. After that, the mechanical performance of the prepared composites was first examined with unconfined and triaxial compressive tests. The results indicated that the Pisha sandstone reduces the composite’s compressive strength. The reduction is caused by the remained polyurethane material on the surface of the remolded aggregate, which reduces its bond strength with the new polyurethane material. Aiming at this issue, this study applied the ethylene-vinyl acetate (EVA) to enhance the bond performance between the polyurethane and remolded sandstone. The test results indicated both the unconfined and triaxle compressive strength of the polyurethane composites were enhanced with the added EVA content. Furthermore, the durability performance of the EVA-modified composites were examined through freeze-thaw and wet-dry cycle tests. The test results indicated the EVA could enhance the polyurethane composites’ resistance to both wet-dry and freeze-thaw cycles. Overall, the modification with EVA can compensate for the strength loss of polyurethane composites because of the applied remolded aggregate and enhance its sustainability.

## 1. Introduction

The demolishing of the aged infrastructure has led to numerous construction waste, which has led to severe environmental burden [1], including the occupation of landfill [2] and pollution of underground water [3]. Recycling of construction waste as aggregate or supplementary cementitious materials is a promising way to resolve environmental burden [4]. Currently, the study on recycling of construction waste has been widely conducted, including the study on the recycled concrete aggregate [5,6], recycled asphalt pavement [7,8], recycled asphalt shingles [9], and etc. Specifically, the recycled cement concrete aggregate has now been applied in field application after detailed investigation, including the application on unpaved road [10], concrete beams [11,12], masonry mortar [13,14], concrete columns [15], and etc. Although currently the study and understanding on recycled polymer concrete aggregate is still quite limited [16]. Specifically, the polyurethane materials have been widely applied to stabilize sandstone for water conservation. However, the aging of polyurethane stabilized sandstone had led to numerous waste polymer concrete and also deteriorated its water conservation ability. This study aims to resolve these issues by recycling the waste polymer concrete as aggregate to re-prepare polyurethane-based composites for sustainable water conservation applications.

Currently, polyurethane is widely applied in construction due to its novel mechanical performance [17], water retention ability [18,19], and proper adhesive performance [20]. Wang et al. [21] immobilized the bacteria with the polyurethane shell to prepare the self-healing concrete. It is found that the higher strength recovery was achieved for the samples with polyurethane immobilized bacteria, compared to that with silica gel immobilized bacteria. The study by Akkoyun and Akkoyun [22] indicated reinforcement with fly ash and blast furnace slag can enhance the tensile strength of the polyurethane composites. Yang et al. [23] examined the performance of polyurethane-based composites with the waste red mud, which can achieve the compressive strength of 38.6 MPa and flexural strength of 12.4 MPa, respectively. It was also found that the polyurethane foam waste can be applied as aggregate and enhance the fatigue performance of the mortar samples [24]. Chen et al. [25] applied the polyurethane to enhance the deicing performance of the asphalt pavement. It was found that the adhesion force to the external ice layer can be significantly reduced with the attached polyurethane layer. The study by Lu et al. [26] supported that the polyurethane-based composites owned better long-term mechanical performance than that of the traditional asphalt concrete. Lu et al. [27] further prepared the pervious pavement with the polyurethane stabilized recycled ceramic aggregate. It was found the prepared samples owned higher compressive strength and rutting resistance compared to that of the normal asphalt concrete. Huang et al. [28] indicated the applied polyurethane can enhance the impact resistance of the subgrade bed under the high-speed railway. The study by Lee et al. [29] indicated the utilization of polyurethane can enhance the damping performance and tensile performance of the polymer concrete prepared with replaced concrete aggregate. Further study by Lee et al. [30] indicated the polyurethane can also enhance the damping property of the prepacked concrete. Junco et al. [31] applied the waste polyurethane foam as an aggregate substitute for mortar preparation. It was found that the polyurethane foam can enhance the workability but deteriorated its alkaline stability. Similar results were also reported in the study by Gadea et al. [31]. Polyurethane [32] has been applied to fix the potholes because of its strong adhesion with the asphalt materials and used as a stabilizer for sandstone [33,34,35]. Despite the wide application of polyurethane as construction materials, the research on recycled polyurethane-based composites is currently still limited.

One major obstacle prohibiting the wide application of the recycled aggregate is that the remained binder on its surface [36] can deteriorate its bonding strength with the new binder materials. Various protocols have been proposed to resolve this issue. One major solution is to conduct the surface treatment for the recycled aggregate to enhance its bonding strength with the new binder material, including surface washing [37] and cement coating [2]. Specifically, the study by Brand and Roesler [38] indicated that surface washing with the acid solution can enhance the bonding strength between the cement matrix and the reclaimed asphalt pavement. Furthermore, it was found that the surface washing could enhance the surface hydrophilicity of the recycled aggregate and thus led to a denser interfacial transition zone between the recycled aggregate and the new binder material [39].

Another type of solution is to modify the binder material for the mixture design optimization. Currently, the addition of superplasticizer [40,41], replacing Portland cement with SCMs [42,43] and adjusting the water/cement ratio [44] have all been conducted for the performance improvement of the concrete with recycled concrete aggregate. Cartuxo et al. [45] applied both the regular and high-performance superplasticizer to adjust the rheological behavior of the concrete with fine recycled concrete aggregate and improve its mechanical/durability performance. The test results indicate the regular and high-performance superplasticizer can increase its 28-day compressive strength by 35% and 63% respectively compared to that of the reference sample. Furthermore, the 90-day shrinkage can be reduced by 2% and 30% by the regular and high-performance plasticizer respectively. Besides that, the study by Brand and Roesler [39] indicated the porous interfacial transition zone along the recycled aggregate can be strengthened with the added silica fume. The compressive strength of the concrete with reclaimed asphalt pavement can thus be enhanced.

Besides the study on bonding performance, the investigation on the durability performance of the concrete with recycled concrete aggregate has also been widely conducted [46]. The freeze-thaw cycle tests indicated the recycled concrete aggregate can deteriorate the freeze-thaw resistance of concrete [47,48,49]. When exposed to freeze-thaw cycles, its inappropriate freeze-thaw resistance can further deteriorate its loading capacity [50], compressive performance [51], seismic performance [52] and cyclic mechanical performance loading capacity [53]. The further study by Salem et al. indicated the recycled concrete aggregate can achieve similar freeze-thaw resistance to normal aggregate under proper air-entraining [54]. Similar results have also been reported in reference [55,56]. The study by Gokce et al. [57] indicated the recycled aggregate from the non-air entrained concrete can deteriorate the freeze-thaw resistance of the concrete. Besides that, the study by Kim et al. [58] indicated replacing cement with waste glass sludge can help to enhance the freeze-thaw resistance of concrete. The study by Farnam et al. [59] indicated that exposing to sodium chloride solution can further promote the development of freeze-thaw damage. Meanwhile, the study on the wet-dry resistance of concrete containing recycled concrete aggregate is relatively limited [60,61]. The study by Zhang et al. [62] indicated the generated thenardite crystals due to wet-dry cycles mainly located in the interfacial transition zone between new cement and the unwrapped surface on the recycled concrete aggregate. The study further supports that the interfacial transition zone between old and new cement demonstrated a more stable performance than that between old and new cement under wet-freeze thaw cycles. The study by Saha et al. [63] indicated the wet-dry resistance of the concrete containing recycled concrete aggregate can be enhanced by replacing cement with fly ash. A similar result was also reported in the study by Qi et al. [64]. On the contrary, the durability study on the concrete with recycled polymer concrete is still limited and the knowledge gap needs to be filled, especially polyurethane polymer-based composites [65,66].

**Figure 1:**
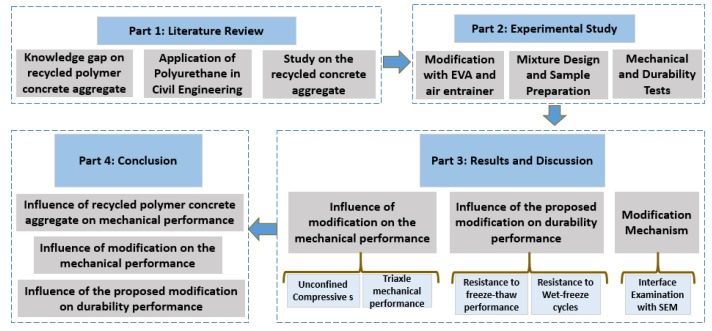
Demonstration for the logic structure of the whole paper.

As mentioned above, currently the investigation and understanding of the recycled polymer concrete aggregate are quite limited. A detailed experimental investigation was conducted in this study to fill this knowledge gap. This study first examined the mechanical and durability performance of the original polyurethane-based composites incorporating the remolded sandstone aggregate. Then the strategy of modifying binder material was adopted for the performance enhancement. The original polyurethane was modified using the EVA content in this study, and the influence of the EVA content on the mechanical performance was then conducted with the unconfined and triaxle compressive tests. Furthermore, the influence on durability performance was also investigated by the wet-dry and freeze-thaw tests. The logic structure of the whole paper is demonstrated in Figure 1. This study can provide a feasible and sustainable protocol for the recycling of remolded sandstone aggregate in the polyurethane-based composites.

## 2. Sample Preparation and Experiment

The raw material for the sample preparation includes the collected Pisha sandstone from the field site and the W-OH type hydrophilic polyurethane. The original Pisha sandstone sample was collected from the Huangpu River Basin, Inner Mongolia, China, where the water erosion and soil loss is a long-term issue. The former studies indicated that the polyurethane could help to stabilize the fragile Pisha sandstone [67,68]. However, the stabilized Pisha sandstone can get aged and cracked due to the insufficient durability of the polyurethane [68], which is called remold Pisha aggregate in this study. The main phase composition of the Pisha sandstone includes quartz, illite, montmorillonite, feldspar, plagioclase, and kaolinite [35]. The as-received Pisha sandstone was further sieved and the samples with the No. 8 sieve size (2.36 mm) were collected for further sample preparation.

**Figure 2:**
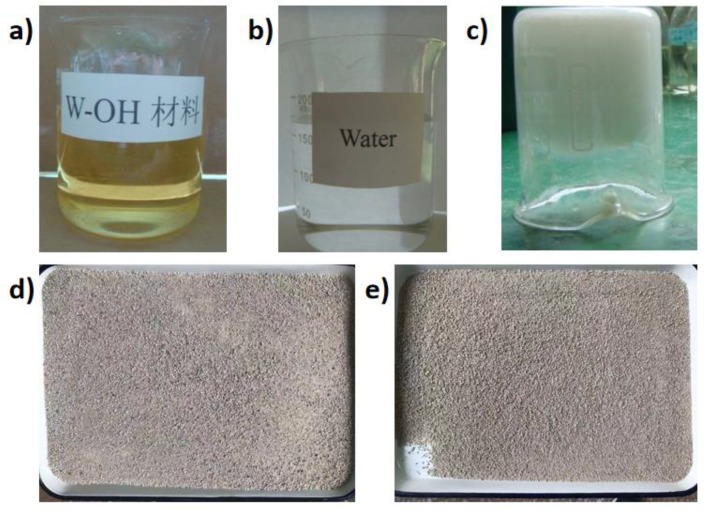
Demonstration of the raw materials for sample synthesis. (**a**) The original W-OH polyurethane primer under oil state; (**b**) tap water to initiate the polymerization process; (**c**) the generated polyurethane polymer; (**d**) the as-received Pisha sandstone aggregate; (**e**) the remolded Pisha sandstone particles with remained polyurethane on the surface.

The W-OH type polyurethane has been used for repairing the concrete materials in the former study [69], which was purchased from the JCK New Material Technology Co. Jiangsu, China. The as-received W-OH polyurethane primer stays in the oil state under the ambient condition as shown in Figure 2a. The W-OH material will get hardened when mixing with water (Figure 2b) and the hardening time can range from 3–15 min based on the polyurethane/water ratio. The hardened polyurethane composite belongs to ductal materials as indicated in Figure 2c.

### 2.1. Preparation and Remolding of the Original Pisha Sandstone/W-OH Composites

The first step is to prepare the polyurethane composites with original Pisha sandstone. The ready-prepared W-OH solution was quickly mixed with the sieved Pisha sandstone (No. 8 sieve size) during the preparation and the mass ratio between the sandstone and W-OH solution is selected to be 4:1 based on the filed experience [67]. To investigate the influence of the W-OH concentration on the mechanical performance of the composites, five different concentration of W-OH solution was used for the sample preparation: 4%, 5%, 6%, 7%, and 8% as shown in Table 1. The mixture of the W-OH solution and the Pisha sandstone aggregate was evenly mixed using the handheld blender for 2 min. Then the mixture was swiftly compacted into the cylinder molds (diameter of 39.1 mm and height of 80 mm) before the hardening of the W-OH polyurethane. The sample was then demolded after 48 h curing.

The next step is to obtain the remolded Pisha sandstone aggregate. The prepared polyurethane composites with original Pisha sandstone first went through 12 wet-dry cycles and then were manually cracked to obtain the remolded Pisha sandstone particles. This process simulated the natural degradation of the polyurethane-based composites and the freeze-thaw cycles were applied based on the protocol shown in Section 2.3. Five types of remolded Pisha sandstone aggregates were then obtained as shown in Table 1. The major difference between the original and the remolded Pisha sandstone aggregate is that the remolded Pisha sandstone was now partially or fully covered with the W-OH polyurethane. The remolded Pisha sandstone aggregate was also sieved to obtain the particles with the size around 2.36 mm for the further sample preparation. The as-received and remolded Pisha sandstone aggregates are shown in Figure 2d,e respectively. It is obvious that more agglomerated Pisha particles were found in Figure 2e because of the binding effect of the remained polyurethane materials.

### 2.2. Preparation and Improvement of the Remold Pisha Sandstone /W-OH Composites

**Figure 3:**
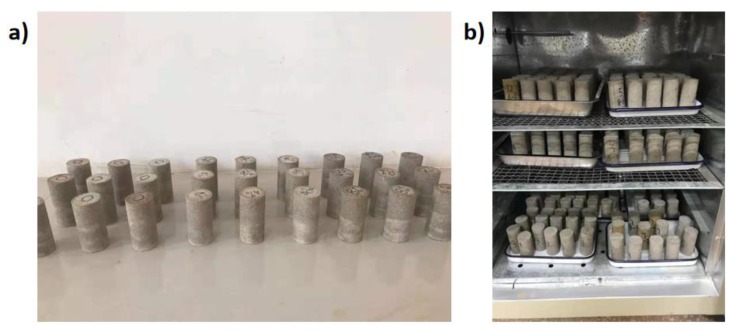
The prepared cylinder samples for the mechanical and durability tests. (**a**) The cylinder samples prepared with original Pisha sandstone/W-OH composites; (**b**) indication of the convection oven for sample drying.

In the field construction, the remolded and cracked Pisha sandstone/W-OH composites are re-solidified with the W-OH polyurethane to resume its water conservation ability [70]. To simulate the re-solidification process, the remolded Pisha/W-OH particles in Table 1 was re-mixed with the W-OH solution to prepare the remolded Pisha sandstone /W-OH composites. Two types of W-OH solution with a concentration of 4% and 8% were applied to re-solidify the remolded Pisha sandstone particles. The cylinder samples as shown in Figure 3a were prepared for further examination, which owns a diameter of 39.1 mm and a height of 80 mm.

As the remained W-OH polyurethane on the surface of the remolded Pisha particle can deteriorate the performance of the remolded Pisha/W-OH composites, the modification on the W-OH was conducted by using ethylene-vinyl acetate (EVA) based on the former research results [69]. Three replacement mass ratios based on the W-OH polyurethane were applied: 5%, 10%, and 20%.

### 2.3. Characterization and Performance Test of the Remolded Pisha/W-OH and Original Pisha/W-OH Composites

The scanning electron microscopy was first conducted to examine the morphology difference between the original and remolded Pisha sandstone particles. The particles were coated with a 20 μm carbon layer before the examination.

Besides the microscale characterization, the macroscale performance evaluation was also conducted, including the unconfined and triaxle compression tests. The unconfined compression test was conducted with the prepared cylinder samples in Section 2.1 and Section 2.2 by using the MTS-CMT5105 universal testing machine. The triaxle compression test was conducted by utilizing the Triaxle shear tester. Three types of confining pressure were applied during the triaxle tests, 50, 100, and 200 kPa. Both the unconfined and triaxle compression tests were conducted based on the standard for soil test method (GB/T50123-1999) [71].

### 2.4. Durability Test of the Pisha Sandstone/W-OH Composites

To better understand the sustainability of the Pisha/W-OH composites in the field condition, the durability tests were conducted for the prepared Pisha Sandstone/W-OH composites, including the freeze-thaw and wet-dry cycle tests. The infrastructure in Inner Mongolia goes through frozen temperatures in the winter and dry conditions in the summer season, respectively. The field examination also indicated the freeze-thaw cycles and wet-dry cycles are the major durability threats to the long-term performance of the infrastructure [72]. Hence these two durability tests were selected for the durability analysis. During the freeze-thaw cycles, the samples were first frozen to −20 °C for three hours and then thawed at 20 °C for another three hours. 80 freeze-thaw cycles were applied to the composites for the durability analysis. During the wet-dry test, the wet cycle was conducted by submerging the polyurethane samples in the water tank with distilled water for 23 h under temperature 20 ± 3 °C. Then the dry cycle was followed by first air-drying the samples for 2 h and then drying the samples in a conventional oven as shown in Figure 3b for another 23 h under 50 °C ± 3 °C. It took 48 h to finish one wet-dry cycle and 12 wet-dry cycles were applied to the composites for the stability evaluation.

## 3. Results and Discussion

### 3.1. Compressive Performance Test of the Original Pisha Stone/W-OH Composites Un

The measured unconfined compressive strength for the five type samples in Table 1 is shown in Table 2. It is clear that the compressive strength will increase with the concentration of the W-OH solution, which is inconsistent with the findings in reference [72]. The increased W-OH concentration will lead to a higher W-OH amount in the composites, which can cover more surface area of the Pisha sandstone aggregate, generate more internal links, and thus lead to higher compressive strength.

**Figure 4:**
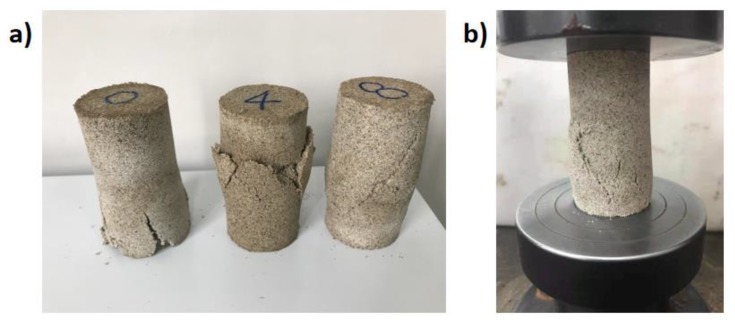
Indication of the samples after compressive strength tests. (**a**) Demonstration of the crushed samples with original (0), R-P-1 (4) and R-P-5 (8) type aggregates; (**b**) test setup for the compressive strength examination.

**Figure 5:**
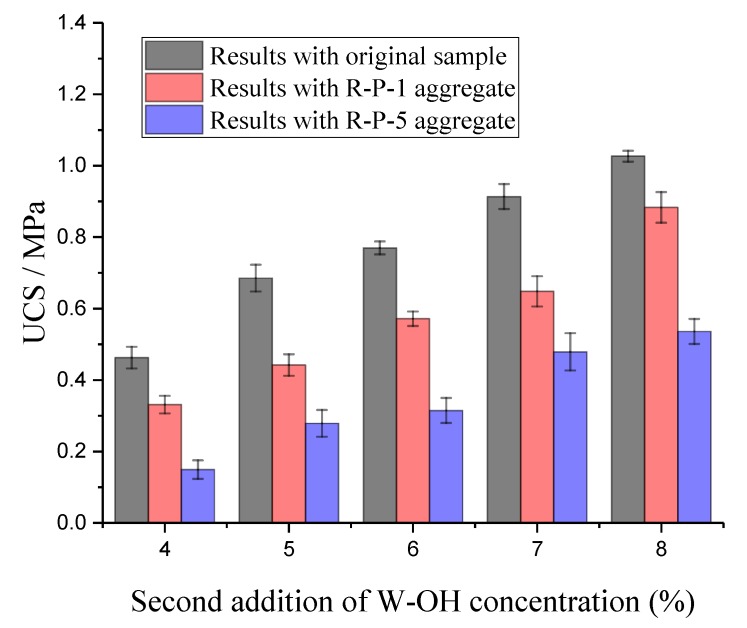
Unconfined compressive strength of the Induration prepared with polyurethane and remolded Pisha sandstone.

The further compressive tests were then conducted with the Remolded Pisha sandstone/W-OH composites. The crushed samples and the test setup are shown in Figure 4a,b respectively. As mentioned in Section 2.2, the composites were prepared with the R-P-1/R-P-5 type remolded aggregate and the original polyurethane. The samples prepared with original sandstone (“0” type sample) were deteriorated by local destruction, while the samples prepared with remolded aggregate (“8” type sample) demonstrate a whole scale shear type failure. The transition on the failure mode is caused by the reduced bond strength because of the remained polyurethane on the aggregate surface. Based on the test results shown in Figure 5, it is clear that the remolded Pisha sandstone will decrease the strength of the polyurethane-based composites. A similar phenomenon has been also reported for the recycled concrete aggregate, where the remained cement paste [73] will decrease the bond strength between the recycled aggregate and the new cement paste. It is assumed that the strength reduction in this study is also caused by the remained polyurethane on the aggregate surface. Specifically, it is obvious that the higher reduction rate was obtained for the R-P-5 type remolded Pisha sandstone aggregate compared to that of this R-P-1 type aggregate. This phenomenon can be caused by that more surface area of the R-P-5 type remolded aggregate (8% W-OH solution) is covered by the remained W-OH. Furthermore, it can be found that the reduction rate will be affected by the concentration of the new W-OH solution for the preparation of remolded Pisha/W-OH composites. Compared to the reference sample, the strength of the R-P-1 type specimen prepared with 4% and 8% W-OH polyurethane solution decreases by 27.9% and 8.4%, respectively. Similarly, the compressive strength for the R-P-5 type composites using 4%, 5%, 6%, 7%, and 8% W-OH solution was lowered by 66.5%, 59.3%, 59.0%, 45.5%, and 47.8%, separately. These results indicated the strength reduction caused by the remolded aggregate can be partially compensated by adjusting the W-OH solution concentration. This can be caused by the mechanism that a higher concentration W-OH solution can help to generate more internal links with Pisha sandstone and lead to higher strength.

### 3.2. Triaxle Mechanical Performance of the Induration Prepared with Polyurethane and Remolded Pisha Sandstone

**Figure 6:**
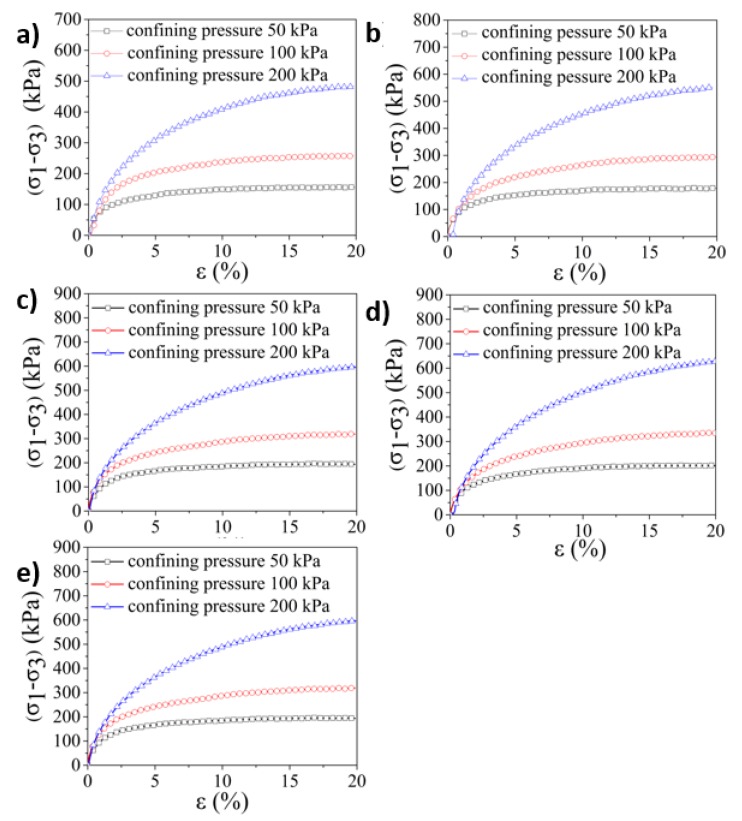
Deviator stress-axial strain curves of remolded Pisha sandstone during triaxle compressive tests. (**a**–**e**) represent the R-P-1, R-P-2, R-P-3, R-P-4, and R-P-5 type remolded aggregate respectively.

**Figure 7:**
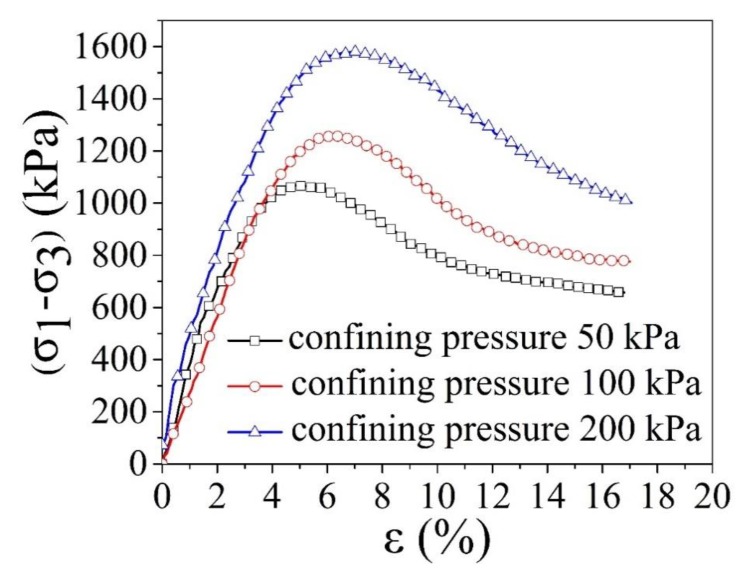
Deviator stress-axial strain curves of original Pisha/W-OH composites during triaxle compressive tests.

The triaxle compressive tests were further conducted with the remolded Pisha sandstone, original Pisha sandstone/W-OH composites, and remolded Pisha sandstone/W-OH composites separately to better understand the compressive performance under confining pressure. The triaxle compressive tests were conducted under the unconsolidated-undrained condition and the confining pressure (50 kPa, 100 kPa, and 200 kPa) was selected based on the local crustal stress condition. The obtained stress–strain relationship for the remolded Pisha sandstone is shown in Figure 6. The clear strain hardening can be observed for all the remolded Pisha sandstone samples as shown in Figure 6, which is inconsistent with the performance of loose sandy soil [74] and indicate the internal connections generated by the W-OH have been almost completely destructed. On the contrary, the straining soft performance can be observed for the original Pisha sandstone/W-OH composites as shown in Figure 7, which is similar to the triaxle property of the soft rock [75]. Furthermore, it is also clear that the failure pressure for all the five types of remolded Pisha aggregate increases with the confining pressure as indicated in Figure 6, which is also caused by the hardening effect of the loose remolded Pisha sandstone. Another observed phenomenon is that the failure stress of remolded Pisha sandstone can increase with its original W-OH concentration. This result can be caused by the enhanced internal friction caused by the remained W-OH polyurethane [76] on the surface of the remolded Pisha sandstone.

**Figure 8:**
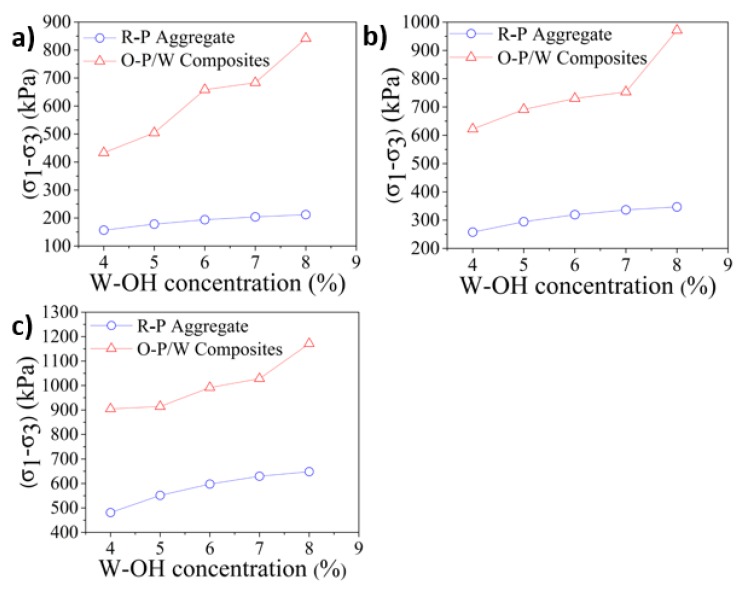
Relationship curve between destructive stress and W-OH concentration under different confining pressures. (**a**–**c**) indicate the results under 50 kPa, 100 kPa and 200 kPa confining pressure.

**Figure 9:**
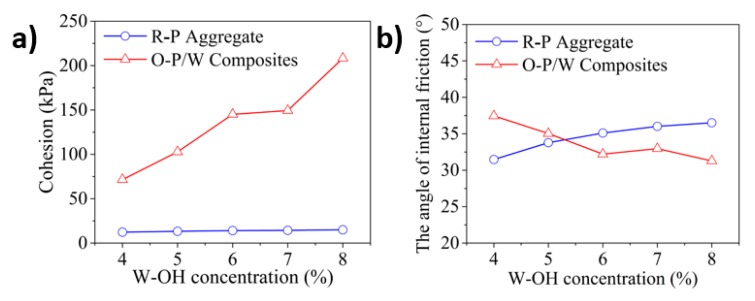
Relationship between cohesion and internal friction angle of remolded solid body and W-OH Pisha sandstone solidified body. (**a**) Demonstration of the measured cohesion for remolded solid body and W-OH Pisha sandstone solidified body; (**b**) Indication of the determined internal friction angle for the remolded solid body and W-OH Pisha sandstone solidified body.

In this study, the stress when the axial strain reaches 15% was defined as the failure stress based on the recommendation in the reference [77]. The comparison of the failure stress between the remolded sandstone and the original Pisha/W-OH composites was then conducted and the results are indicated in Figure 8. At the confining pressure of 50 kPa, the failure stress of the original Pisha sandstone/W-OH composites increases from 433.7 kPa at 4% W-OH concentration to 843.7 kPa at 8% W-OH concentration. This result is in accordance with the research findings based on unconfined compressive strength shown in Table 2. Furthermore, the reduction of failure stress because of remolding was 64.2% and 75.2% respectively for the 4% and 8% W-OH specimen at the confining pressure of 50 kPa. The confining pressure also significantly affects the failure pressure. Under the confining pressure of 200 kPa, the failure stress of original Pisha sandstone/W-OH composites increases from 904.3 kPa at 4% W-OH concentration to 1171.3 kPa at 8% W-OH concentration. It is clear that the increased confining pressure will lead to higher failure stress and relative lower strength reduction ratio. A more detailed study was conducted to determine the cohesion strength and friction angle based on the Mohr-Coulomb failure criterion [78] and the obtained results are shown in Figure 9. The cohesion strength for the original Pisha/W-OH composites at 4%, 5%, 6%, 7%, and 8% W-OH concentration were 71.9 kPa, 101.1 kPa, 145.4 kPa, 148 kPa, and 208.9 kPa respectively. The related reduction rates on cohesion strength caused by remolding increase from 83.0% at 4% W-OH concentration to 92.9% at 8% W-OH concentration respectively. The increase of the strength reduction rate is because more internal links will be broken during remolding for the samples prepared with a higher concentration of W-OH solution. Meanwhile, the friction angle for the remolded samples increases from 31.4° at 4% W-OH concentration to 36.5° at 8% W-OH concentration. The increase is caused by the enhanced internal friction because of the higher amount remained W-OH material [76] on the surface of the remolded Pisha aggregate.

### 3.3. Mechanical and Durability Examination of the Composites Prepared with Remolded Pisha Sandstone and EVA Modified W-OH Material

The results in Section 3.2 indicated that the remolding can almost completely break the internal links in the Pisha/W-OH composites and significantly reduce its triaxle compressive strength. Furthermore, the data in Section 3.1 demonstrated that the utilization of remolded Pisha sandstone can reduce the compressive strength of the polyurethane composite. To resolve this issue, the modification of the original polyurethane was conducted to enhance its internal bonding with the remolded Pisha sandstone. Based on the former research experience [69], the modification with EVA was conducted to enhance the bonding performance between W-OH and remolded Pisha sandstone.

**Figure 10:**
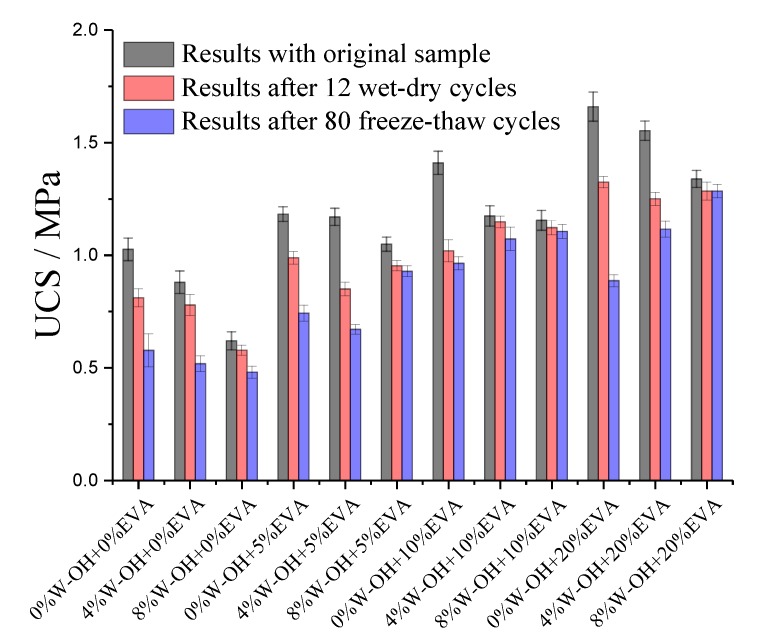
Strength of the Induration prepared with EVA modified polyurethane and remolded Pisha sandstone (0%W-OH, 4%W-OH, and 8%W-OH represent the Original, R-P-1 and R-P-5 type aggregates respectively; 0% EVA, 5% EVA, 10% EVA and 20% EVA represent the Original, 5% EVA, 10% EVA and 20% EVA modified W-OH Polyurethane were applied to re-solidify the remolded Pisha sandstone).

The examined compressive strength results for the samples re-solidified with EVA modified W-OH is demonstrated in Figure 10. It is obvious that the modification with EVA can significantly enhance the unconfined compressive strength of the composites containing remolded Pisha sandstone. The data are shown in Figure 10 demonstrated under 5% EVA content, the compressive strength for the polyurethane composites containing original Pisha aggregate, R-P-1, and R-P-5 type remolded aggregates are 1.18, 1.17 and 1.04 MPa respectively, which are 107.5%, 106.4%, and 95.4% compared to that of the reference sample (the composites prepared with 8% original W-OH polyurethane and original Pisha sandstone as indicated in Table 1). Furthermore, the samples prepared with 10% EVA modified W-OH can fully compensate for the strength loss because of the remolded Pisha sandstone (106.8% and 105.0% for R-P-1 and R-P-5 type remolded aggregate respectively).

Besides the performance evaluation on mechanical performance, further studies on the durability performance of the EVA modified W-OH composites were also conducted. Based on the local climate condition in Inner Mongolia, the durability performance under wet-dry and freeze-thaw cycles were examined. The evaluation was conducted by comparing the unconfined compressive strength before and after the durability tests as shown in Figure 10. The results indicated the lowest strength reduction was achieved by the composites with 10% EVA modified W-OH. The strength for the samples containing R-P-1 and R-P-5 type remolded aggregate were reduced by 2.21% and 2.86% respectively by the 12 wet-dry cycles, and 8.69% and 4.24% separately by the 80 freeze-thaw cycles as shown in Figure 9. Although higher strength was achieved for the samples with 20% EVA modified W-OH composites, the better durability performance was achieved by the samples with 10% EVA modified W-OH. The results are because of the observed phenomenon in the former reference that the higher EVA concentration can lead to agglomeration in the W-OH materials [69]. The agglomeration will lead to uneven distribution of mechanical properties and thus cause stress concentration under the recycled environmental or mechanical loading [79], which can then lead to reduced durability performance.

**Figure 11:**
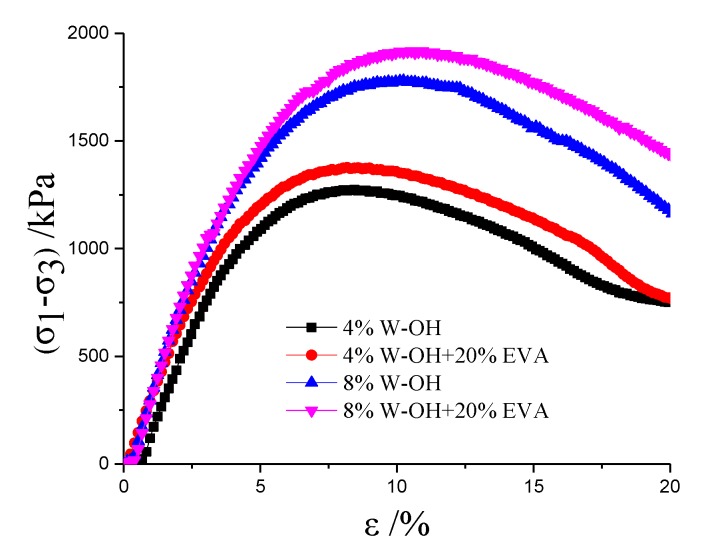
Strength of the induration prepared with EVA modified polyurethane and remolded Pisha sandstone.

Furthermore, the triaxle compressive tests were conducted for the EVA modified W-OH composites. The obtained results are shown in Figure 11. It is clear that the added EVA can also enhance the triaxle compressive strength of the W-OH composites, which is inconsistent with the unconfined compressive strength. Specifically, it should be noticed that the samples with remolded Pisha aggregate has higher triaxle compressive strength compared to the composites with original Pisha aggregate as shown in Figure 11. This is caused by the higher friction angle of the remolded Pisha aggregate as shown in Figure 9.

### 3.4. Constitutive Analysis for the Remolded Pisha Sandstone Composites

To better understand the influence of confining pressure and remolding on the stress–strain performance of the composites, the Duncan–Zhang model was applied for the constitutive analysis of the Pisha sandstone composites as shown in Equation (1).

(1) $$\sigma_{1}-\sigma_{3}=\frac{\varepsilon_{1}}{a+b\varepsilon_{1}}$$

where the parameters a, b represent the soil properties and confining pressure, *σ*_3_ is the confining pressure value (kPa), and *ε*_1_ is the axial strain.

**Figure 12:**
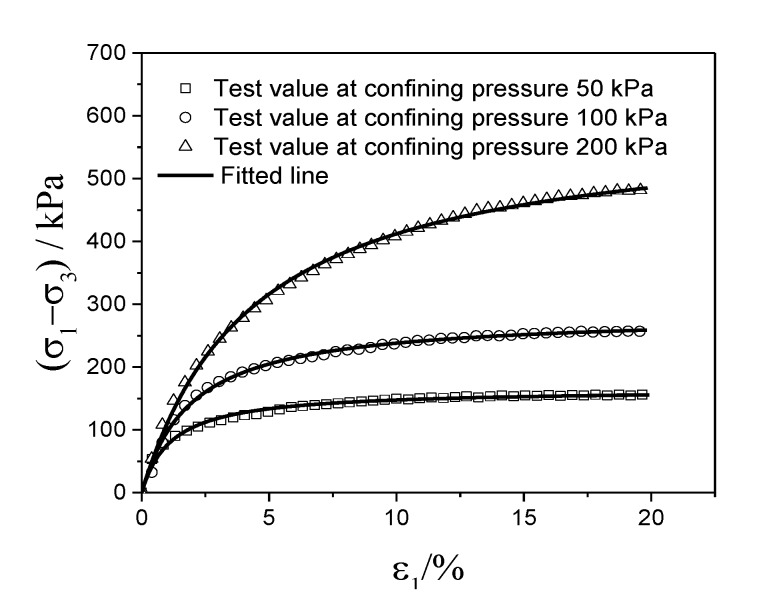
Constitutive analysis for the remolded W-OH Pisha sandstone composited with the W-OH concentration of 4%.

One example of the fitted results based on Equation (1) is demonstrated in Figure 12. It is clear that the test curve and the fitting curve are in good agreement. The correlation coefficients for all the fitted samples are all above 0.97, which further supports the compatibility of the Duncan-Zhang model.

It can be known from Equation (1) that when *ε*_1_→∞, (*σ*1 − *σ*3)_ult_ can be represented with 1/b. The destruction ratio *R*_f_ can be further defined as shown in Equation (2). According to the relevant literature [80], the destruction ratio *R*_f_ is generally between 0.5 and 1. In summary, the destruction ratio *R*_f_ of remolded solidified body, the model parameters a, b, the ultimate deviatoric stress and the destructive stress are shown in Table 3. The fitted failure stress is in accordance with the analysis in Section 3.3.

(2) $$R_{f}=\frac{{\left(\sigma_{1}-\sigma_{3}\right)}_{f}}{{\left(\sigma_{1}-\sigma_{3}\right)}_{\mathrm{ult}}}$$

### 3.5. Morphology Characterization of the Original/Remolded Pisha Sandstone and Pisha/W-OH Composites

**Figure 13:**
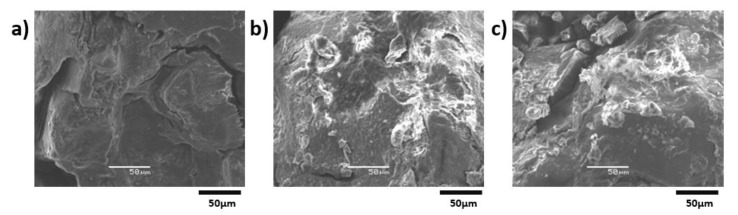
Demonstration of the remolded Pisha sandstone solidified with W-OH type polyurethane. (**a**) The original Pisha sandstone; (**b**) R-P-1 type remolded Pisha sandstone (4% W-OH); (**c**) R-P-5 type remolded Pisha sandstone (8% W-OH).

**Figure 14:**
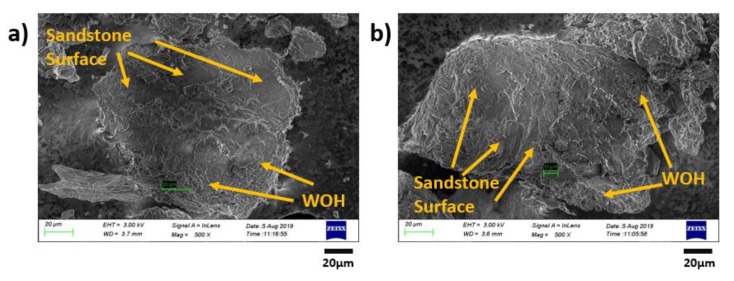
Demonstration of the aggregate in the composites prepared with remolded Pisha aggregate (R-P-1 Type) and original polyurethane with 8% concentration. (**a**) and (**b**) Indication of the local structure for the composites prepared with remolded Pisha aggregate (R-P-1 Type) and original polyurethane with 8% concentration.

**Figure 15:**
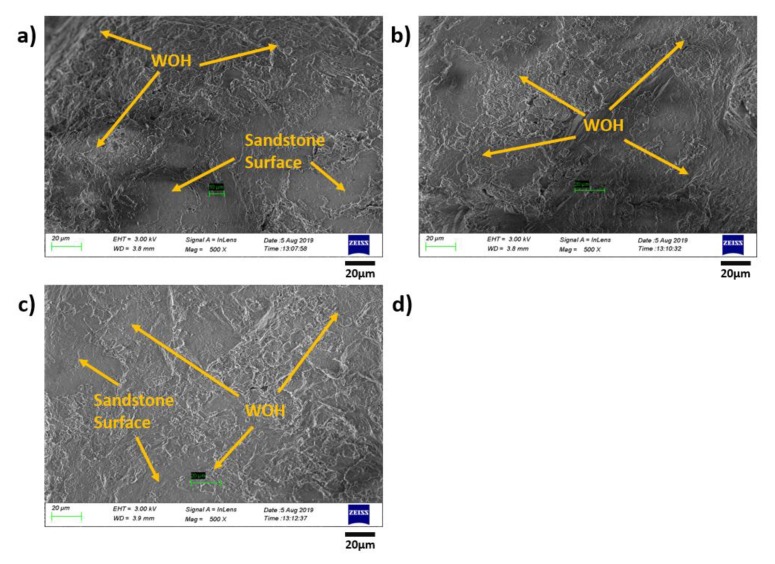
Demonstration of the aggregate in the composites prepared with remolded Pisha aggregate (R-P-1 Type) and 20% EVA modified polyurethane with 8% concentration. (**a**–**c**) Demonstration of the local structure for the composites prepared with remolded Pisha aggregate (R-P-1 Type) and 20% EVA modified polyurethane with 8% concentration.

Further studies were conducted for the morphology analysis of the original and remolded Pisha aggregate. The examination was conducted with SEM and the results are shown in Figure 13. It is clear that the typical layer structure for the sandstone [81] can be observed in Figure 13a. The W-OH and sandstone can be clearly separated by the surface morphology difference, which has also been adopted in reference [82]. The area covered by the remained W-OH increases with the W-OH concentration can be found for the remolded sandstone aggregate shown in Figure 13b,c. The poor adhesion between the remained W-OH and new W-OH materials can lead to the decreased performance of the remolded Pisha/W-OH composites. Similar issues were also reported for the recycled concrete aggregate with remained hydrated cement on the aggregate surface [83,84]. Compare to the R-P-1 type aggregate, the higher surface area in the R-P-5 type aggregate is covered by the remained polyurethane, which can explain the difference in strength reduction rate shown in Figure 5. The morphology analysis was further conducted for the remolded aggregate re-solidified with original W-OH and EVA modified W-OH respectively. The related results are shown in Figure 14 and Figure 15 respectively. It is clear the added EVA can enhance the surface area covered by the W-OH as shown in Figure 14 and Figure 15. These results support the assumption that the EVA can lead to the higher internal bond between the remolded Pisha aggregate.

## 4. Conclusions

This study investigated the mechanical and durability performance of the polyurethane composites with remolded sandstone aggregate. To compensate for the strength loss due to the added remolded aggregate, the EVA content was applied for the modification of the W-OH polyurethane. Then the unconfined and triaxle compressive tests were conducted to evaluate the mechanical performance tests. The durability under wet-dry and freeze-thaw cycles were also investigated. The major conclusion of this study are shown below:

(1) The remained polyurethane material on the surface of the remolded sandstone aggregate can enhance its internal friction. The samples with a higher amount of remained polyurethane can have higher compressive strength under the triaxle test.
(2) The remained polyurethane material can reduce the unconfined compressive strength of the polyurethane composites containing remolded aggregate. The reduction is caused by the poor bonding between the remained polyurethane on the remolded aggregate surface and the new polyurethane material. The strength loss can be partially compensated with a higher concentration of polyurethane solution.
(3) The modification with EVA can fully compensate for the strength loss of the polyurethane-based composites due to the applied remolded sandstone aggregate.
(4) The EVA content can enhance the resistance to both freeze-thaw and wet-dry resistance of the polyurethane-based composites. The optimized EVA content based on mechanical and durability performance is 10m% based on the polyurethane content.

In the future, the research team aims to further apply the research findings in field construction to resolve the environmental burden because of the accumulated remolded Pisha sandstone and improve its resistance to environmental loadings. Furthermore, the field examination will help to identify the possible limitation of the proposed protocol and point out further research needs.

## Figures and Tables

**Table 1:** Preparation of the Pisha sandstone composites with original polyurethane.

Sample Type	O-P/W-1	O-P/W-2	O-P/W-3	O-P/W-4	O-P/W-5
Aggregate	Original Pisha Sandstone around 2.36 mm				
W-OH concentration	4%	5%	6%	7%	8%
Remolded Aggregate Type	R-P-1	R-P-2	R-P-3	R-P-4	R-P-5

**Table 2:** Relationship between the W-OH concentration and the unconfined compressive strength of the original Pisha stone/W-OH composites.

Sample Type	O-P/W-1	O-P/W-2	O-P/W-3	O-P/W-4	O-P/W-5
Strength (MPa)	0.47	0.69	0.76	0.91	0.10

**Table 3:** Demonstration of the fitted parameters based on the Duncan–Zhang model.

*σ*_3_/kPa	W-OH Concentration/%	a	b	(*σ*_1_ − *σ*_3_)_ult_ /kPa	(*σ*_1_ − *σ*_3_)_f_ /kPa	R*_f_*	The Average Value of R*_f_*
50	4	0.00739	0.00601	166.39	153.78	0.941	0.93
50	5	0.00555	0.00533	187.62	175.44	0.957	
50	6	0.00543	0.00484	206.61	192.23	0.940	
50	7	0.00624	0.00461	216.92	198.97	0.934	
50	8	0.00597	0.00442	226.24	207.56	0.879	
100	4	0.00683	0.00352	284.1	251.56	0.906	0.90
100	5	0.00677	0.00306	326.8	284.8	0.906	
100	6	0.00561	0.00287	348.43	308.26	0.932	
100	7	0.00689	0.00266	375.94	320.58	0.874	
100	8	0.00792	0.00254	393.7	325.95	0.880	
200	4	0.00729	0.00169	591.72	459.56	0.813	0.80
200	5	0.00765	0.00142	704.22	518.13	0.777	
200	6	0.00653	0.00135	740.74	560.12	0.807	
200	7	0.0074	0.00122	819.67	583.66	0.768	
200	8	0.00449	0.00129	775.2	629.2	0.817	
